# The national portfolio of learning for postgraduate family medicine training in South Africa: experiences of registrars and supervisors in clinical practice

**DOI:** 10.1186/1472-6920-13-149

**Published:** 2013-11-08

**Authors:** Louis Jenkins, Bob Mash, Anselme Derese

**Affiliations:** 1 Division of Family Medicine and Primary Care, Stellenbosch University and Western Cape Department of Health, George Training Complex, George, South Africa; 2 Centre for Education Development, Faculty of Medicine and Health Sciences, Ghent University, Ghent, Belgium

**Keywords:** Portfolio, Family medicine, Postgraduate, Adult learning, Work-based, Supervision, Service pressure

## Abstract

**Background:**

In South Africa the submission of a portfolio of learning has become a national requirement for assessment of family medicine training. A national portfolio has been developed, validated and implemented. The aim of this study was to explore registrars’ and supervisors’ experience regarding the portfolio’s educational impact, acceptability, and perceived usefulness for assessment of competence.

**Methods:**

Semi-structured interviews were conducted with 17 purposively selected registrars and supervisors from all eight South African training programmes.

**Results:**

The portfolio primarily had an educational impact through making explicit the expectations of registrars and supervisors in the workplace. This impact was tempered by a lack of engagement in the process by registrars and supervisors who also lacked essential skills in reflection, feedback and assessment. The acceptability of the portfolio was limited by service delivery demands, incongruence between the clinical context and educational requirements, design of the logbook and easy availability of the associated tools. The use of the portfolio for formative assessment was strongly supported and appreciated, but was not always happening and in some cases registrars had even organised peer assessment. Respondents were unclear as to how the portfolio would be used for summative assessment.

**Conclusions:**

The learning portfolio had a significant educational impact in shaping work-place based supervision and training and providing formative assessment. Its acceptability and usefulness as a learning tool should increase over time as supervisors and registrars become more competent in its use. There is a need to clarify how it will be used in summative assessment.

## Background

South Africa has seen major advances in healthcare to address its quadruple burden of disease; namely HIV and tuberculosis; non-communicable chronic diseases; injury and violence; and maternal, neonatal and child health [1]. The national plan for re-engineering primary health care (PHC) emphasizes the central role of the family physician as a clinical leader in the district health team. The planned national health insurance scheme needs a massive scaling up of the numbers of doctors [2,3]. South Africa was short of 80 000 health care professionals in 2008 [4]. The challenge of training and keeping sufficient numbers of competent doctors in all 52 health districts is influenced by multiple factors, including career choices, job satisfaction, career advancement, work conditions, and educational opportunities [4,5]. The importance of social accountability requires that education and training of health professionals must be aligned with the health needs of the country [6]. The national human resource policy aims for 900 family physicians by 2020, which will require a doubling of the number of registrars in training from 2014 [7].

**Figure 1:**
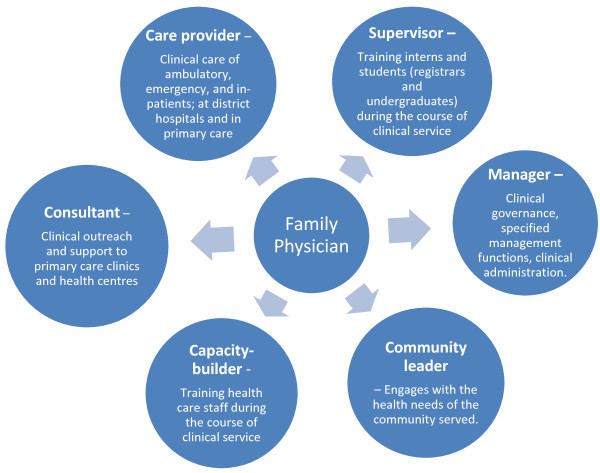
**The competencies expected of a South African family physician [**[11]**].**

National training outcomes and a single national exit examination have been developed for family medicine [8-10]. Registrars enter a 4-year programme at one of the eight university departments, attached to a clinical complex consisting of PHC facilities, a district hospital, and a regional hospital. Eligibility for the exit examination of the national College of Family Physicians, to qualify as a consultant family physician, requires completion of three years of supervised clinical training in a registrar post in one of these complexes and submission of a portfolio of learning with satisfactory evidence of learning. Figure 1 illustrates the various competencies expected from the family physician [11].

While workplace-based assessment (WPBA) has been discussed in educational policy in South Africa for the last 20 years, most postgraduate programmes still examine their registrars away from the PHC and district context, usually in simulated environments at the university [12,13]. Worldwide, the growing interest in quality improvement and increasing demands for social accountability have shifted the focus of assessment from the university to the work place [14-16]. WPBA typically involves the use of tools for direct observation of patient encounters or procedures, 360-degrees peer review, and significant event analysis [14,17].

Portfolio-based learning as part of registrar’s WPBA encapsulates many aspects of competency-based assessment and has been introduced in many countries and disciplines in the last 20 years [18]. We can define the portfolio as “a collection of material made by a professional that records and reflects on key events and processes in that professional’s career” [19]. Many purposes for keeping a portfolio exist, which must be made explicit to both the registrars and their supervisors [18,20]. Depending on the purpose, the portfolio’s content could range from a logbook-type enumeration of skills performed to a personal journal with evidence of deep reflection. It could be for personal or professional development, for curriculum requirements or to satisfy external agencies, such as the College of Family Physicians.

The portfolio needs to go beyond being just a collection of achievements and demonstrate reflective understanding of how and why these achievements contributed to personal and professional growth. In other words, reflective learning, as part of lifelong learning, embedded in everyday professional practice, is an integral part of the portfolio [21,22]. Attaining this deeper level of reflection on learning is not supported by the pedagogic framework of teacher-centred (as opposed to learner-centred) education people grew up with, or by the service delivery workload which squeezes out time to reflect in- or on-action [23]. The process of reflection itself requires engagement in critical self-awareness, with skills needed in mindful practice [24,25]. Therefore the creation of an ideal portfolio constitutes a shift in one’s educational paradigm, which must be achieved in addition to understanding the portfolio requirements. For this type of learning to happen as part of the process of keeping a portfolio, the registrar needs support from a skilled supervisor, who understands reflective learning [18].

Since the 1970s adult learning, or andragogy, introduced the concepts of self-directed learning, accumulated experience as a resource for learning, and problem-based, real world learning [26]. Experiential learning describes how registrars learn from having a particular experience, reflecting on that experience, developing abstract conceptualisations and then testing these in a new situation [27]. Facilitating such experiential learning should be an extension of everyday life, and as valid as other forms of learning [18]. This form of deep learning, as opposed to lecture style didactic surface learning, assumes intrinsically motivated registrars, actively involved in their own learning, exploring their thinking in learning conversations with supervisors and others [28-30].

The competencies expected of family medicine registrars in South Africa are contained in the five national unit standards for the discipline as follows [10]:

1. Effectively manage him/herself, his/her team and his/her practice, in any sector, with visionary leadership and self-awareness, in order to ensure the provision of high-quality, evidence-based care

2. Evaluate and manage patients with both undifferentiated and more specific problems cost-effectively according to the bio-psycho-social approach

3. Facilitate the health and quality of life of the family and community

4. Facilitate the learning of others regarding the discipline of family medicine, primary health care, and other health-related matters

5. Conduct all aspects of health care in an ethical and professional manner

The portfolio is a tool to facilitate learning and attainment of these outcomes in the clinical context. Typically, a reflection on a patient encounter, significant event analysis or a direct observation of a patient encounter by the trainer will raise multiple complex issues. Issues may relate to clinical care, the health system, relationships, teamwork, personal growth or ethics. All of these dimensions could be captured in the portfolio. Although portfolio ownership rests with the registrar, learning through experience, reflection, and discussion can only take place effectively with adequate support and focussed time [31]. This applies to both formative and summative assessment and emphasises the importance of training clinical supervisors, and giving them feedback from registrars [32-35]. Registrars must be coached in reflective practice and this must be embedded within their training. This asks for a shift from supervision, where the registrar is being watched, to training, where the concept of journeying together is stronger, to eventually mentoring, where both trainer and registrar reflect on their own journeys. While flexibility is part of the strength of the portfolio, a basic structure is important for review and assessment of the content [18]. Consequently, the South African portfolio contains in its basic structure the following sections [36]:

1. Introduction and purpose of the portfolio

2. Learning outcomes expected

3. Learning plans, reflections on rotations and supervisor reports

4. Educational meetings

5. Direct observations of consultations, procedures, and teaching events

6. Written assignments

7. Logbook

8. Emergency medicine training certification

9. Additional courses and conferences

10. Final assessment

Pre-printed tools to assist the registrars and supervisors and space to give feedback or grade the registrar are also incorporated into the portfolio. While the summative assessment of the portfolio will always contain subjectivity, the use of a portfolio assessment tool, with grades for every section, and a final overall grade for the portfolio, serves three functions:

1. The portfolio grade can count towards the university’s assessment of clinical family medicine in the Masters programme.

2. A satisfactory completed portfolio over three years is mandatory for the national College exit examinations.

3. It encourages the registrars to regularly reflect on and document their learning, prompts the learning process, and through changed behaviour leads to better patient care, and a habit of lifelong learning and reflection.

The portfolio was recently introduced at a national level in South Africa, and still fits like “new shoes” which must be worn in [37,38]. While much has been written on using portfolios in postgraduate training and assessment, the practical use of portfolios, particularly in South Africa, is still not well understood. The aim of this study was to explore the views of registrars and supervisors regarding the portfolio’s educational impact, acceptability, and perceived usefulness for assessment of competence.

## Methods

### Study design

This was a prospective, descriptive study, using qualitative semi-structured interviews with key informants.

### Ethical considerations

The study was carried out in compliance with the Helsinki Declaration and approved by the Health Research Ethics Committee of the University of Stellenbosch, with reference number N09/10/258.

### Setting

The eight medical schools in South Africa each have a postgraduate family medicine training programme, offered at a Master’s level (MMed), over 4 years. Various combinations of clinical rotations exist in PHC, district hospitals, and specialist departments in regional hospitals. The registrars work under direct supervision of either family physician consultants or specialists in the regional hospitals. Supervisors and most programme managers also do clinical work. With the recognition of the specialty of Family Medicine in 2007, the College of Family Physicians has introduced a unitary exit examination for family medicine training in the country. A satisfactory portfolio of learning over three years is required for their Fellowship of the College of Family Physicians [FCFP (SA)]. National consensus was reached in 2010 on the content and construct validity of the portfolio and a draft national portfolio implemented [38]. After a national survey to obtain feedback on the portfolio it was further refined and the final portfolio is now the standard in all eight programmes [36,39].

### Researchers’ relationship to the topic

Taking a reflexive stance, we need to give a brief explanation of how the researchers are positioned contextually in relation to this research [40]. The first author (LJ) has been working in clinical practice for 20 years. He is a family physician supervisor of registrars in training and the training complex co-ordinator for the George training complex under Stellenbosch University. He is therefore immersed in the everyday issues of clinical work, training, learning, and assessment of registrars. The second author (BM) is currently the Head of the Division of Family Medicine and Primary Care at Stellenbosch University and is responsible for the final approval of portfolios for entry to the FCFP (SA) national exam. He previously developed the postgraduate training programme at Stellenbosch University and the previous logbook, aspects of which were incorporated into the portfolio. He has a strong background in qualitative research and has published extensively in this area [41-45]. Until recently he was a full time family physician working and teaching in clinical practice in South Africa. The third author (AD), from Belgium, brings an external European perspective to the study and helped to make explicit the differences between clinical learning and assessment in the South African context versus the European context. He has extensive experience in using portfolios in the workplace and is an academic and practicing family physician. He is also familiar with the local context through his involvement over several years in a project to develop family medicine education in South Africa that was funded by the Flanders Interuniversity Council [46,47]. This is the third article, in a series looking at the development of the family medicine learning portfolio in South Africa, by all three authors.

### Study population and sampling

Nine registrars and eight supervisors from all eight universities were purposively sampled as key informants because of their experience with using the new portfolio during the previous year (see Table 1). The first and second authors are well acquainted with all eight university programme managers. We asked the programme managers to recommend registrars and supervisors who were using the portfolio regularly and who would be willing to give an in-depth account of their experience. Participants were then approached according to these recommendations.

**Table 1:** Study participants

	**University**	**Registrar**	**Family physician supervisor**
1	Cape Town	1	1
2	Stellenbosch	2	1
3	Free State	1	1
4	Pretoria	1	1
5	Witwatersrand	1	1
6	Limpopo	1	1
7	Natal	1	1
8	Eastern Cape	1	1

### Data collection

Telephonic in-depth interviews lasting 30-60 minutes were conducted by the principal investigator. We used an interview guide (see supplementary files) to conduct the interviews and the topics to be explored were selected from the literature review and our own previous survey of registrars’ and supervisors’ experiences [39]. The interviews were digitally recorded and the interviewer made field notes during the interviews. The opening question was, “How are you experiencing the portfolio in your context?” In line with the study objectives the three main issues that were explored included how the portfolio contributed to learning, how its practical use could be improved, and how it could be used in assessment of competence. From these, the purpose of the portfolio, experience of its use in clinical learning, balance of work and learning, personal development, formative versus summative assessment, supervisor meetings, practicalities of secure academic time, use of portfolio tools, and ways of ensuring regular entries and progress, were discussed.

### Data analysis

We used ATLAS.ti version 6.2.27 software and the 'framework analysis’ approach described by Ritchie and Spencer [48]. 'Framework analysis’ is an analytical process which involves five distinct yet highly interconnected stages. These stages are: familiarization; identifying a thematic index; coding; charting; mapping and interpretation. Familiarisation with the data involved reading the transcripts in their entirety several times and checking against the audio tapes, as well as reading the field notes taken during the interviews. During this process codes began to emerge and led to the development of a thematic index. The thematic index was deductively structured according to the objectives of the study, but the codes within this structure emerged inductively from the data. Following this all the transcripts were coded according to the thematic index. The fourth stage created charts that collated together all the data on each of the three objectives. Data for supervisors and registrars was charted separately. These charts were then used for interpretation of the data in terms of the range and strength of different viewpoints and possible associations between them [48-50].

Respondent validation was enabled by giving feedback on the provisional analysis to registrars and supervisors in subsequent workshops, which involved groups of between 30 and 40 registrars and supervisors at all eight universities. During these workshops participants had the opportunity to confirm, clarify or modify the interpretation of the results. Furthermore we also presented our work to the heads of departments and programme managers, some of whom are supervisors themselves, at the eight universities.

## Results

The results are presented according to the three objectives for the study that explored the portfolio’s educational impact, acceptability and use in assessment.

### Educational impact

#### Portfolio as a learning tool

The portfolio was seen as a useful tool to capture how the registrar learns, thinks and practices. However, it not only captured the registrar’s learning, but through its requirements ensured that there was more attention given to the registrar’s learning in the workplace and thus became a tool that stimulated supervision and educational activities. It required them to draw up a learning plan, organize, and audit their own learning. The need for regular meetings and engagement with their supervisor was explicit from the beginning, when the registrar drew up his or her learning plan, as well as during subsequent reviews of progress:

“…So the registrar gets to a rotation, and then they are supposed to do the learning plan. That I think is incredibly useful, because they put what they know about the topic, what they want to learn, and how they’re going to learn it. Then I meet with them again at the middle of the rotation, and when we look at the learning plan, which they often haven’t actually done, but often they have and discussed it with the site facilitator, then we look at the reflection…” (Supervisor)

In terms of facilitating the registrar’s learning the portfolio required a certain amount of time committed to educational meetings and examples of case discussions or significant event analysis were often mentioned. These were not just captured in the portfolio, but provided the impetus for these discussions with their supervisors:

“We discuss some of the patients that come out of the portfolio, or the situations that comes from it…it becomes part of the afternoon’s discussion, because these are difficult patients, difficult scenarios. So that’s why sometimes the portfolio translates into a learning tool, which I don’t think is just documentation and evidence type tool, but also actually a learning tool.” (Supervisor)

“It becomes a learning tool, making learning (and work) easier, for example discussing difficult patient scenarios in an educational meeting.” (Supervisor)

#### Registrars’ personal engagement with their learning

The extent to which the portfolio portrayed a comprehensive picture of the registrar’s development was related to how much the registrar took ownership of and engaged with their portfolio:

“*The portfolio is not “another project” to be handed in. Ownership sits with the registrar. It is their learning journey, their journal to keep. It should not be in the possession of faculty. It should show how the registrar is learning and developing to become the person he or she wants to be.” (Supervisor)*

For registrars to really capture their learning in the portfolio respondents highlighted the need to be more organised in setting aside time to complete the portfolio, as well as the need for greater awareness of when their clinical experience was part of their learning, the ability to reflect on this experience and conceptualise what they had learnt:

“…A mental note, yes. I do make lots of mental notes. We are supposed to submit our portfolios at the end of the month. It’s not up to date, because… I don’t know, I can’t blame anyone for that. I think it’s just me meaning to do that, and then you’re just tired and you forget. Then later on, maybe when it’s going to be your mid-block assessment, the mid-block, then you quickly write it up, and remember what you meant to write there. So I don’t think that one is a problem with the portfolio more than just me not being organised and on top of it…” (Registrar)

“…the people learn an incredible amount. When you talk to them you can hear this very clearly, you know it, but they don’t catch it” (Supervisor)

“Actually we are almost testing something else. It is almost something more than the portfolio. It is a person’s discipline and planning skills…” (Supervisor)

With the portfolio concept, and even adult learning, still in its infancy, registrars and supervisors simply were not aware of or experienced with reflective thinking and writing. Although reflection was happening, it was almost unconscious, and very seldom documented:

“I battle to get a reflection from them, in the portfolio. Where we do get a reflection, is during the learning conversation, but otherwise, I battle to get anything meaningful from them, that is documented in the portfolio. When you talk to (the registrars), you realize that they have actually reflected on this, but it is not structured, and they have not even realized that they have been reflecting…” (Supervisor)

Registrars were reluctant to document their learning needs, making it difficult to compare subsequent portfolio entries with previous ones, in order to get a picture of their development. This reluctance may have stemmed from a sense that one should not reveal any weaknesses, deficiencies or mistakes in one’s portfolio and should only include evidence of competency rather than learning. The impression was that the portfolio was another project or task to hand in, rather than a learning tool:

“…then he said now he needs to rewrite this. Then I said no, you should not rewrite this. This is the proof of what we learnt together today, and if you hand this in like this, anyone looking at this will see there was a thorough discussion, writing and learning took place. He went and rewrote it, everything he learnt, on a clean sheet of paper, and this is not really what we want, I think…” (Supervisor)

While some registrars were negative about the portfolio, many more were positive. They felt that the portfolio made visible the translation of theory into practice, simply by writing down their reflective thinking.

“…yes, and the Calgary-Cambridge communication model, what is great is that I can ask someone in the clinic to quickly sit in and listen…just take this marking sheet in the portfolio and just give me a score, or even just a global score, of what you think of my consultation skills. Or you can record your own consultations, which are what I have done a few times now, and then go over them again at home and rate myself in the portfolio…” (Registrar)

#### Supervisor engagement

Participants reported that there were too few family physician supervisors for the number of registrars. Most supervisors were not in joint staff positions (appointed to both university and department of health) and therefore battled to prioritise their training role and to balance their service delivery and training responsibilities. Many supervisors did not have the skills to facilitate reflection, give useful feedback, or adequately assess registrars. There was a need expressed among registrars to have better role models, to have regular supervisory meetings, and to receive more feedback:

“…It would have been nice to actually train under them, under family physicians who will actually apply the management that is expected of us in an everyday, on an everyday basis to patients that you have. But we don’t have models…”(Registrar)

“…so it was literally a month where it was quite a struggle to see a consultant, let alone to now get them to sit down and listen to you tell them about a patient…” (Registrar)

While supervisors other than family physicians are often not engaging the registrars, a few were providing opportunities to observe and record registrar learning. A rotation like surgery became meaningful with a good supervisor:

“…I’m enjoying surgery now because I’m with someone who really enjoys teaching, and even if you belong to his team, he doesn’t mind if you want to learn other skills. So, I suppose it depends on who you are with…” (Registrar)

The portfolio was acting as a catalyst to *“…force a meeting with the supervisor…”.* This was due to the requirement for reports from the supervisor, their signatures to verify entries and recording of direct observations. Registrars found the need for signatures negative. While ownership of learning and portfolio completion rests with the registrar, a theme of shared accountability with the supervisor emerged. This clearly indicated that unless the supervisor was held accountable for their role as clinical trainer in terms of regular direct observations and educational meetings, the culture of prioritising service delivery continued to overshadow learning.

### Acceptability

#### Overwhelming service demands

A major theme was that clinical service continually overwhelmed efforts to organize learning, reflection and writing. There was also a mismatch between the educational outcomes, which reflected a more ideal reality that we should strive towards, and the actual reality of overworked health workers struggling to survive in a tough environment. When working in the regional hospital the registrar might also be providing services that do not have relevance to their training as a family physician:

“…we do tough work here and survival. A lot of the time they’re in survival mode, and I think that’s why Kleinman, Arthur Kleinman, he said the worst people to learn from are registrars because they’re in survival mode…” (Supervisor)

“…It’s all about service, it’s all about the number of patients you’ve seen, and filling in of forms and things like that. For instance, we are being taught assessment, we are being taught the principles of medicine, ethics and that kind of thing. We are being taught things that we are unable to actually execute when you are expected to see 40 patients, and as inexperienced as I am. So I wouldn’t be able to spend the time as I am expected to spend with a patient and in that small space of time, I must have done all those things and seen 40 patients…”(Registrar)

“…It depends on the department you are in. In theory it works because you are able to get there and say these are the outcomes, these are the things that you need when you are there. But you don’t find the same enthusiasm in all the departments, if you know what I mean. What happens is that in some departments you are seen as another workforce. As long as the department is run. As long as you do the ward work, as long as you are in theatre, that kind of thing. Do you understand what I mean? Even if your needs are not really met…” (Registrar)

In terms of secure academic time, many programme managers had created regular opportunities for the registrar and supervisor to meet and discuss the registrar’s learning. Typically an afternoon was set aside every 2 weeks for case-based discussions, critical incident reviews, or review of an article:

“we have now always on the second Tuesday afternoon created a session, or an opportunity, a routine, that the registrar and his supervisor can meet for an hour to discuss the learning process, to talk about learning, and I think this is meaningful.” (Supervisor)

#### Stressful work-learn dichotomy

The dichotomy between working and learning created particular stress. Sometimes the modules and tasks required by the academic programme were incongruent with the registrar’s clinical context and experience. This was particularly true when registrars were rotating through a regional hospital department that did not share the context of family medicine practice. In this situation they became almost “lost” in that clinical specialty, and tended to lose regular contact with their family physician supervisor or overall coordinator. Sometimes the concurrent requirements of the academic programme were not incorporated into their personal learning plans for the workplace environment:

“…but the clinical modules that we’re busy with, we don’t necessarily come home and study that every day. We kind of come home and have to do online stuff if you’ve got an assignment due this week…” (Registrar)

“the consultation module was really very nice…you learn so much more about how to communicate with a patient, how to exchange information…I can really say that I apply it much more in my day to day working environment. Ethics was also great to do (as a module), because I had no idea about ethics. But it feels to me that it was something I had to do because it was not part of my learning plan. I think this is where it will stay…” (Registrar)

#### Logbook limitations

The logbook, which documented competency in a list of clinical skills, was perceived as very limited, in need of revision and conversion to an electronic format. Some respondents wanted more detail on the number of times a skill was performed. Registrars were not always sure how much of the assessment of their competency in clinical skills was a self-assessment or an assessment by their supervisor, and how this related to the few skills that were directly observed and scored in another section of the portfolio. There was also confusion as to how the logbook documented the development of skills over time as opposed to confirming that competency had been achieved. There was a sense that the logbook reflected the minimum required, and could be expanded, even with a view to continuing into one’s future career:

“…The log book is actually incredibly limited, but I say that’s just the core. Write down everything that you actually do, and when you go to a job and you say I’ve got a special interest in anaesthetics, show them all the more complicated anaesthetic procedures you’ve done…” (Supervisor)

#### Having learning tools at hand

While participants were agreeable with the current paper-based portfolio, there was a call for more electronic tools to support it and ultimately an electronic portfolio. People did not read the guide to the portfolio, or felt it was not clear, and asked that it be made more user-friendly. Organization of the portfolio was also viewed as important. Most agreed that observed consultations were important, but difficult to do. Capturing assessment of procedural skills, having case-based discussions, and capturing significant event analysis were all experienced as difficult to achieve. Some suggestions to overcome the difficulty of capturing learning in the workplace included having the learning tools daily at hand, regularly updating the portfolios, preferably electronically, and a central coordinator who collates portfolio entries monthly and warns the registrars early when they fall behind. If the portfolios were completed as expected, it would give valuable feedback to the service and the programme and may eventually influence the learning environment constructively:

“…it may even be, in the long run, a formative process to the other specialities. ..Exactly, exactly. You know, and we did have feedback saying gee, that really made me think of something different. That really did help, that really was helpful. So obs and gynae, I think, have been quite good with that.…” (Registrar)

### Assessment

Participants expressed uncertainty as to how the portfolio contents would be summatively assessed. Respondents felt that most summative assessment was focusing on completeness of the portfolio, while the next step would be to look at the quality of the portfolio entries.

“…it is also valid that you just submit your work. This is already a big step, because it provides evidence for what has been done. But the next step is to decide on the quality of that evidence…” (Supervisor)

Regular meetings between the registrar and the supervisor were essential to assess learning and provide formative feedback:

“…it gives us a chance to touch base both with me and the site coordinator or the specialist. So it’s like three people looking at where we’re going. What’s the current situation and what’s happening.”(Supervisor)

While there was strong support for an end-of-rotation summative assessment, a mid-rotation formative assessment with the supervisor, to look at progress, was also valued:

“So that’s the mid-block assessment, and then I ask the supervisors to do an observed - no, what’s it called - a continual assessment. Often they haven’t done one up till then, but then that gives them a chance to give the registrar feedback.” (Supervisor)

“And then you might make any changes to the plan, and then at the end of the block I see them again, and then we do an exam using the observed consultation, one or two consultations. Psychiatry we only do one, but it’s 45 minutes, and another continuous assessment from the consultants.” (Supervisor)

The value of this interaction with and involvement of the supervisor, who sees how the registrar performs and confirms it with a score and feedback in the portfolio, cannot be overemphasized:

“…The one I have had was quite helpful because it kind of forces you to look at how you were before you came to the department and how far you have come, and what more do you still have to learn. So in paediatrics for me it was very helpful, very, very helpful. I think generally it is the few who take time to do it…” (Registrar)

With the shortage of family physicians and other identified supervisors in the country, some registrars had taken more ownership of their learning, and initiated peer assessment:

“We have planned to actually try and do it amongst ourselves, because we know what is expected. We know what’s expected of us in terms of holistic approach to a patient, …..so you actually just know about them during the exam, your end of block exam. It then ends up being something that you fake for them…Like if I’m on first call for instance, that is what we’ve decided on doing. If I am first call and I’m free and I can come to the clinic, so we mark each other. So there it’s the same mark sheet that is used for our exams, and then we just randomly pick a patient and then I will be the examiner and then my other colleague is my examiner as well. So that’s a habit, because at the usual clinics where we are, you don’t even get all these assessments. I don’t know, it’s the old ways that everyone is doing, and no one is supervising you…” (Registrar)

The tension between summative and formative assessment was well recognized by most participants. Considering the purpose of the portfolio, they felt that we need to develop summative assessment indicators that assess the formative aspects captured in the registrar’s portfolio of learning.

## Discussion

### Educational impact

Registrars found the portfolio useful to plan and organize their learning. While the portfolio at this stage is mostly a collection of learning activities, its educational impact could be enhanced through increasing the registrar’s awareness of learning opportunities and their ability to reflect on these experiences. The portfolio has made the challenges of introducing competency-based adult learning more visible and made explicit the need for a supportive learning climate within the clinical environment [37,51]. Critical to prioritizing learning within the pressurized clinical service was a shared accountability between the registrar and the supervisor, with registrars taking responsibility for their own learning and supervisors providing regular feedback. The need for direct observations and case-based discussions in educational meetings made regular registrar-supervisor meetings obligatory. There was a clear need for supervisors to improve their mentoring skills and for registrars to fully embrace adult learning. Service pressure made it difficult to reflect on work and document learning. Nevertheless, this demanding clinical context in which the portfolio of learning is embedded is very rich in terms of experience and can provide fertile soil from which the habit of lifelong learning can grow [23]. This worldwide challenge is exacerbated in low and middle income countries where human resources are scarcer and workload is very high. Training programmes in this context need to anticipate this and make plans to overcome this challenge to the use of portfolios [52-54].

A review of the educational impact of portfolios concluded that “improved student-tutor relationships” was one of the main benefits, together with increased self-awareness and engagement in reflection [55]. Introducing the portfolio may be an intervention that stimulates a shift in supervisory style from directing to guiding and from counting to reflecting. It may in fact help to create the very learning environment that it is meant to be documenting, becoming itself an agent of change. This educational relationship between the registrar and supervisor within the context of a community of practice is perhaps the most vital and difficult area to navigate [56]. Worldwide, but particularly in low and middle income countries such as South Africa, there are too few effective supervisors who understand their role as trainers and clinical role models and who are recognized and rewarded accordingly. Good doctors do not necessarily have skills in teaching, giving feedback, or assessment [33]. Teaching registrars involves vulnerability, relationship, honesty, trust and kindness [33]. Incentives to encourage and reward mentors could include that mentors are kept “in the know” with developments in medicine and medical teaching, build better relationships with registrars, receive feedback themselves, and meet potential future family physician colleagues [35].

Within our culture of service delivery, clinicians have an established culture of documenting their clinical reasoning in patient notes. What is also needed is a culture of professional development in which clinicians capture their learning in a clear, concise, continuous way possibly using a portfolio [23,35,56,57]. It was evident that self-management skills that build reflection and develop resilience are much needed for both registrars and supervisors [23,58,59].

### Acceptability

The paper-based portfolio was accepted, albeit with a call for more electronic tools that are compatible with mobile devices, and eventually an electronic portfolio, similar to examples from more developed countries [35,60,61]. Completing a logbook in the portfolio created a particular conflict between the traditional counting of procedures performed and the need to reflect on and learn from one’s performance. Personal organization of learning, self-management and a discipline of regularly updating the portfolio were strongly supported [18]. Observed consultations and procedures were important, but difficult to do in the workplace [52,62]. Suggestions to make this easier included having learning tools daily at hand, regular portfolio updates, and a central coordinator who collates portfolio entries monthly and gives feedback. Regular face-to-face meetings between registrars and supervisors ensure authenticity of learning with supervisors’ signatures, the absence of which is a disadvantage in pure e-portfolio systems [35].

### Assessment

While assessment at this stage focusses on completeness, for example the number of direct observations and educational meetings, the next step is to assess the quality of learning.

There was strong support for both an end-of-rotation summative and a mid-rotation formative assessment. The shortage of supervisors has led some registrars to initiate peer-assessment. Within family medicine, as the discipline has become more established, there has been a shift to train and assess more in the district hospitals and PHC, with less exposure to regional hospital departments [4,11]. Such a shift requires that a culture of training and assessment is initiated, valued and nurtured by both the district health services and universities.

Current forms of assessment encourage registrars to demonstrate their competence, whereas valuable learning is often based on mistakes, errors, problems and less than perfect outcomes [63,64]. Demonstrating learning also requires evidence of a shift in competency over time from a less than adequate starting point. This has implications for how the summative assessment of the portfolio is constructed. Summative assessment of the portfolio should focus on whether these formative activities have taken place adequately. The supervisor and registrar are best able to determine the quality of learning and progress as documented in the portfolio, while the programme co-ordinator is best able to determine the completeness of all that is required. We can think of this as assessment-in and assessment-on the portfolio, not unlike reflection-in and reflection-on action [23].

Registrars and supervisors spoke of a 'work-learn’ dichotomy whereby they struggled to integrate the theory of best practice in terms of the consultation, ethics or evidence-based medicine into everyday work. Part of the challenges of work-based assessment is integrating course assignments with work assessment, assessing how doctors actually practice [65,66]. As assessment drives learning, a grade and formative feedback provide measurement and meaning to work-based learning, encouraging the registrar to develop into a family physician [67]. Indirectly a good portfolio also provides assessment and feedback on the training programme itself in the local context and can be valuable to the programme manager.

Although the use of portfolios for work-based assessment is becoming best practice internationally it needs to be tailored to the contextual realities of low resourced settings where it may be seen as an additional burden for registrars already on the edge of burnout or depression [68-70]. Unlike tertiary health centres, the district health services are not used to the demands of speciality training in South Africa. The portfolio requirements make visible the expectations on registrars and supervisors and brings into focus the need to integrate cultures of service and learning rather than allowing them to be perceived as if they are in opposition [4,6].

#### Limitations

This study sought to understand the experiences and opinions of registrars and supervisors who had used the portfolio in South Africa. We purposefully recruited appropriate participants who would give rich information from across South Africa to get a broad perspective of experience, representing all postgraduate programmes in the country. This meant that because of travelling distances of up to 1400 kilometres we decided on telephonic interviews. We acknowledge that telephonic interviews may have limitations compared to face-to-face interviews, for example periods of silent reflection could be more acceptable in face-to-face interviews. However, because the author was familiar with the context of the training programmes and many of the key people, he was able to engage the respondents easily and encourage them to elaborate on their answers. The interviews also lasted between 30-60 minutes, which was deemed sufficient for thorough exploration of the key topics. The results of the study as with all qualitative research cannot be easily generalised to other populations and the readers will need to decide what findings are transferable to their own context.

#### Recommendations

The following recommendations can be made from the findings:

1. Continue with the national portfolio as an acceptable tool to support work-based learning and assessment.

2. Advocate for a culture of clinical training in the health districts, recognizing the co-benefits of service delivery and clinical learning.

3. Develop registrars’ and supervisors’ self-awareness and ability to reflect on and learn from their clinical experience in a structured way that can be documented.

4. Develop electronic tools and move towards an e-portfolio.

5. Focus on developing the capacity of supervisors to support adult learning in the work place and to formally recognise their role as trainer or mentor.

6. Allow time for new educational practice to be integrated into the work-place with a shift from traditional pedagogy to adult learning.

7. Develop an approach to summative assessment of the portfolio. A portfolio assessment tool will be described and evaluated in a future study.

## Conclusions

The portfolio primarily had an educational impact through making explicit the expectations of registrars and supervisors in the workplace. This impact was tempered by a lack of engagement in the process by registrars and supervisors who also lacked essential skills in reflection, feedback and assessment. The acceptability of the portfolio was limited by service delivery demands, incongruence between the clinical context and educational requirements, design of the logbook and easy availability of the associated tools. Its acceptability and usefulness as a learning tool should increase over time as supervisors and registrars become more competent in its use. The use of the portfolio for formative assessment was strongly supported and appreciated, but was not always happening and in some cases registrars had even organised peer assessment. There is a need to clarify how it will be used in summative assessment.

## Competing interests

The authors declare that they have no competing interest.

## Authors’ contributions

All authors contributed to the planning and design of the study. LJ conducted the interviews. All authors analysed the results and contributed equally to the final writing of the article. All authors read and approved the final manuscript.

## Pre-publication history

The pre-publication history for this paper can be accessed here:

http://www.biomedcentral.com/1472-6920/13/149/prepub
